# Can we make it up? - second-look surgery due to post-operative residual tumour in patients diagnosed with diffuse glioma

**DOI:** 10.1007/s11060-026-05489-4

**Published:** 2026-03-03

**Authors:** Sebastian Jeising, Johannes Reinken, Marion Rapp, Michael Sabel, Franziska Staub-Bartelt

**Affiliations:** 1 Institute of Neuropathology, Heinrich-Heine-University Düsseldorf, Düsseldorf, Germany; 2 Department of Neurosurgery, Uniklinikum Erlangen, Friedrich-Alexander-Universität Erlangen-Nürnberg, Erlangen, Germany; 3 Medical Faculty, Heinrich Heine University, Düsseldorf, Germany; 4 Brain Cancer Centre, Beta Clinic Bonn, Bonn, Germany; 5 Department of Neurosurgery, University Hospital Giessen, Giessen, Germany

**Keywords:** Diffuse glioma, Surgical re-intervention, Residual tumour, Extent of resection, Survival

## Abstract

**Purpose:**

Complete resection (CR) of contrast-enhancing (CE) and non-contrast-enhancing (nCE) tumour compartments is a key prognostic factor in diffuse gliomas. However, despite an intraoperative impression of CR, early postoperative MRI may reveal residual tumour. This study evaluated outcomes of patients undergoing early second-look surgery for unplanned residual tumour volume.

**Methods:**

Patients undergoing surgery for diffuse gliomas between 2013 and 2023 were screened for surgical re-intervention within six weeks after initial resection. Patients undergoing early second-look surgery due to unplanned residual tumour on postoperative MRI were included. Volumetric MRI analyses, RANO resection classification, functional neurological outcomes, perioperative complications, and survival were assessed.

**Results:**

Among 1.558 glioma patients (CNS WHO grade 2–4), 447 underwent multiple surgeries, of whom 46 received second-look surgery for residual tumour. Resection status shifted from 80.4% submaximal after first surgery to supramaximal or maximal resection in 86.96% after second-look surgery. Residual tumour volumes were significantly reduced for both T1-CE and T2-nCE components (*p* < .001). Functional and neurological status remained stable (KPS and NIHSS, *p* > .5). In newly diagnosed glioblastoma patients (*n* = 28), RANO class 1 after second-look surgery was associated with longer overall survival compared to RANO 2B (13.8 vs. 8.0 months; *p* = .043). The 2- and 3-year OS rates were 33.34% and 16.67% in RANO class 1, while no patients in RANO class 2B survived beyond 2 years.

**Conclusion:**

Early second-look surgery for unplanned residual tumour enables a high rate of (supra-)maximal resections without compromising functional outcomes and may improve survival in selected patients.

## Introduction

Over the past two decades, the role of neurosurgical resection in the treatment of diffuse glioma has been substantiated by multiple landmark studies. In glioblastoma (GBM), complete removal of contrast-enhancing (CE) tumour tissue has been shown to improve progression-free survival (PFS) [1, 2]. More recent evidence indicates that resection of non-contrast-enhancing (nCE) tumour beyond a residual volume of < 5 cm³ further prolongs both PFS and overall survival (OS) [3, 4]. Additionally, several studies have demonstrated that upfront surgical resection confers a significant survival benefit compared with a watch-and-wait strategy in patients with lower-grade diffuse gliomas [5]. However, the optimal extent of resection (EOR) remains less well defined [6]. Yet, increasingly more data suggest that supramaximal resection of FLAIR-hyperintense tumour regions may delay malignant transformation and improve survival [7–9]. Given the long survival trajectories in this patient group, however, avoidance of permanent neurological deficits remains a fundamental priority [10, 11].

Intraoperative delineation of tumour margins is challenging due to diffuse infiltration into surrounding tissue. A variety of adjunct technologies have been introduced to improve visualisation, including intraoperative ultrasound (ioUS) [12], neuronavigation [13], fluorescence-guided surgery with 5-aminolevulinic acid (5-ALA) [1], and intraoperative MRI (ioMRI) [14]. Beyond this, the EOR is critically influenced by tumour location in eloquent areas and by intraoperative neurophysiological findings [15, 16], which ultimately shape the surgeon’s perception of *resectability* [17, 18].

Glioblastoma recurrence is inevitable and typically occurs after a median of nine months. Also, at recurrence, complete re-resection plays a pivotal role in disease management and may even compensate for an incomplete initial surgery (Ringel 2016). Honeyman et al. demonstrated that removal of > 95% of tumour volume or < 2.25 cm³ of residual CE tumour after re-resection is prognostic for survival (Honeyman 2024). Since the EOR is usually determined within 24–48 h postoperatively [11], neurooncological neurosurgeons are sometimes confronted with an important dilemma: can unexpected residual tumour be safely managed by early re-resection?

To address this question, the present retrospective study evaluates patients diagnosed with diffuse glioma who underwent unplanned, early *second-look surgery* for residual tumour following the initial resection.

## Methods

### Patients

This study is a retrospective single-centre cohort analysis conducted at a university neuro-oncology referral centre. Patients undergoing surgery for diffuse glioma between 2013 and 2023 at the Department of Neurosurgery, University Hospital Düsseldorf, were screened for second-look surgery, defined as surgical re-intervention within ≤ 6 weeks of initial resection due to residual tumour. The institutional database included 1.558 surgically treated glioma patients, of whom 447 underwent at least one re-operation. Among these, 46 patients received early re-resection within 6 weeks after initial surgery due to unexpected residual tumour on postoperative MRI. The decision to perform early second-look surgery was made in an interdisciplinary neuro-oncology tumour board or, in selected urgent cases, by the responsible consultant neurosurgeon after review of early postoperative MRI findings. No predefined volumetric threshold was applied; decisions were based on resectability, eloquence, patient performance status, and anticipated surgical safety. Functional status was assessed using the Karnofsky Performance Score (KPS) and neurological status using the National Institutes of Health Stroke Scale (NIHSS). Progression-free survival (PFS) and overall survival (OS) were defined as time to radiological progression and death, respectively. Neuropathological diagnosis and WHO grading followed the classification valid at the time of surgery; GBM diagnoses in long-term survivors within RANO class 1 were confirmed according to the 2021 classification of tumours of the CNS [19].

### Extent of resection

Assessment of the EOR was based on early postoperative MRI. The imaging protocol included gadolinium contrast-enhanced T1-weighted (T1-CE) sequences, T2/FLAIR for non-enhancing (T2-nCE) components, and diffusion-weighted imaging. Tumour volumetry was conducted using the Brainlab Elements platform (Brainlab GmbH, Munich, Germany). The SmartBrush tool enabled semi-automated region-growing segmentation of pre- and postoperative tumour compartments (CE and nCE) and resection cavities. Residual tumour volume was measured in cubic centimetres for CE and nCE compartments. In addition to raw volumetric measurements, resections in the GBM subgroup were classified according to the RANO resect classification system, validated as a prognostic marker in both primary and recurrent GBM: RANO class 1: “supramaximal CE resection” (0 cm3 CE + ≤ 5 cm3 nCE), class 2: “maximal CE resection” (2 A: “complete CE resection” (0 cm3 CE + > 5 cm3 nCE); 2B: “near total CE resection” (≤ 1 cm3 CE)), class 3: “submaximal CE resection” (3 A: “subtotal CE resection” (≤ 5 cm3 CE); 3B: “partial CE resection” (> 5 cm3 CE)), class 4: “biopsy” (no reduction of tumour volume) [4, 20].

### Statistical analyses

All statistical analyses were performed using IBM SPSS Statistics (Version 29.0; IBM Corp., Armonk, NY, USA). Quantitative data were assessed for normality using the Shapiro–Wilk test and were non-normally distributed; therefore, all analyses used non-parametric tests. Paired continuous variables were compared using the Wilcoxon signed-rank test, and independent continuous variables using the Mann–Whitney U test. Categorical variables were analysed using the χ² test or Fisher’s exact test, as appropriate.

Categorical variables are presented as counts and percentages, referring to available data points. PFS and OS were estimated using the Kaplan–Meier method, and survival differences were assessed with the log-rank test. All tests were two-tailed, and *p* < .05 was considered statistically significant.

All graphics (e.g., violin and scatter plots) were generated in Python (Google Colab, Version 3.9) using matplotlib and seaborn. Sankey diagrams were created with SankeyMATIC (www.sankeymatic.com), and schematic illustrations with BioRender.com.

### Ethics approval

This retrospective study was approved by the ethics committee of Heinrich-Heine-University Düsseldorf (Study-ID: 2025–3212).

## Results

### Overview

Between 2013 and 2023, 1.558 patients received brain tumour surgery at the Department of Neurosurgery at Düsseldorf University Hospital and were diagnosed with diffuse glioma. 447 underwent more than one surgery. Excluding postoperative complications requiring surgery as well as tumour biopsies only, 82 patients underwent two tumour resections within 42 days (6 weeks). 46 of those underwent unplanned *second-look surgery* as post-operative MRI had revealed residual tumour.

All second-look resections followed contemporary principles of glioma surgery. Standard adjuncts included neuronavigation, intraoperative neurophysiological monitoring (IONM), awake cortical and subcortical mapping when indicated, fluorescence-guided surgery using 5-ALA, and ioUS. Early postoperative MRI was obtained at a median of 2 days postoperatively (IQR 1–3), with 52.2% of patients (24/46) imaged within the first 48 h. Adjuvant therapy followed contemporary EANO guidelines [11, 21, 22].

The mean age of the cohort was 59.0 ± 13.1 years, with a female-to-male ratio of 1:1.09. Second-look surgery was performed predominantly after initial glioma surgery (82.6%), with a mean interval of 10.4 ± 6.4 days between procedures. Mean postoperative residual tumour volume after the first surgery was 2.67 ± 0.51 cm.

According to the WHO classification valid at diagnosis, tumours were classified as grade 4 in 80.4% (all glioblastomas, *n* = 37), grade 3 in 15.2% (*n* = 7), and grade 2 in 4.3% (*n* = 2). MGMT promoter methylation was present in 45.9% of glioblastomas. All grade 2–3 tumours were IDH-mutant, including three with 1p/19q codeletion (Fig. 2A).

Adjuvant therapy for glioblastomas after first diagnosis (*n* = 28) consisted of standard radiochemotherapy according to the *Stupp protocol* [23] in 89.3% (*n* = 25) of patients, temozolomide monotherapy in one patient, and radiochemotherapy combined with Lomustine chemotherapy (CCNU) according to the *Herrlinger protocol* [24] in two patients. The median interval between the first surgery and initiation of adjuvant therapy was 37.5 days (IQR: 31.3–43.3).

### Localisation and surgical strategy

**Fig. 1:**
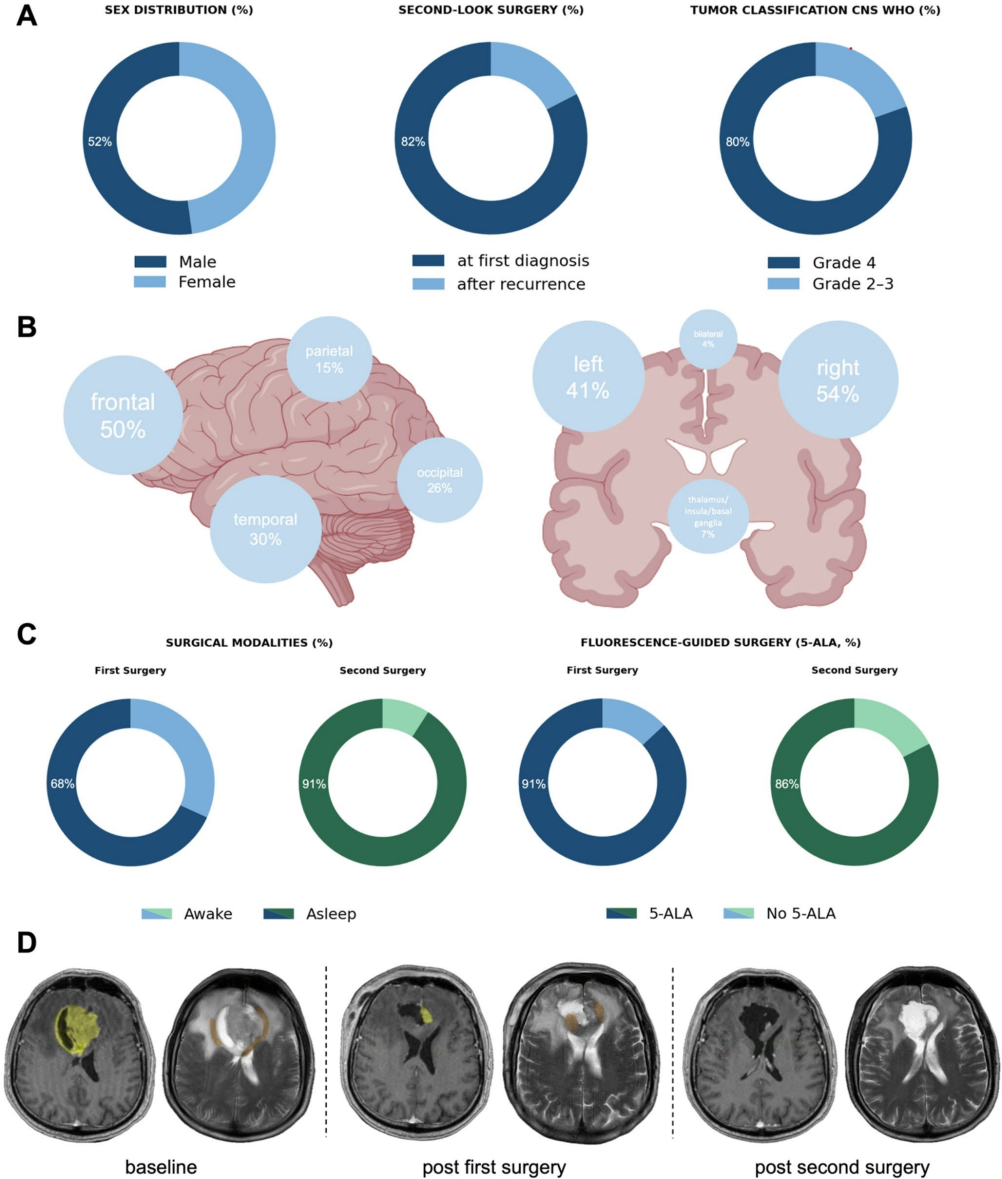
Characteristics of the patient cohort. **A**: Sex distribution, time point of surgery (initial vs. recurrence), and tumour classification within the cohort, **B**: tumour localisation in the included patients, **C**: surgical strategy including use of awake and fluorescence-guided surgery. **D**: MRI from a representative case with residual T1-CE and T2-nCE tumour volumes after first surgery. From left to right T1-CE and T2-nCE baseline to post-second surgery. Postoperative MRI demonstrating T1-CE (yellow) as well as T2-nCE (orange) residual tumour. MRI after the second surgery shows supramaximal resection. Figure 1B was created using BioRender.com

Tumours were localised in motor or speech eloquent areas in 39.13% (*n* = 18). Tumour localisation, time point of surgery, as well as tumour classification, sex distribution and a case sample are illustrated in Fig. 1.

Surgical strategy (Fig. 1C) comprised awake surgery in 31.81% (first vs. second *n* = 14/4), use of IONM in 97.73% (*n* = 43/42) and fluorescence-guided surgery using 5-ALA in 86.96% of the cases (*n* = 40/*n* = 38). All resections were conducted by specially trained consultant neurooncological neurosurgeons.

### Resection results

**Fig. 2:**
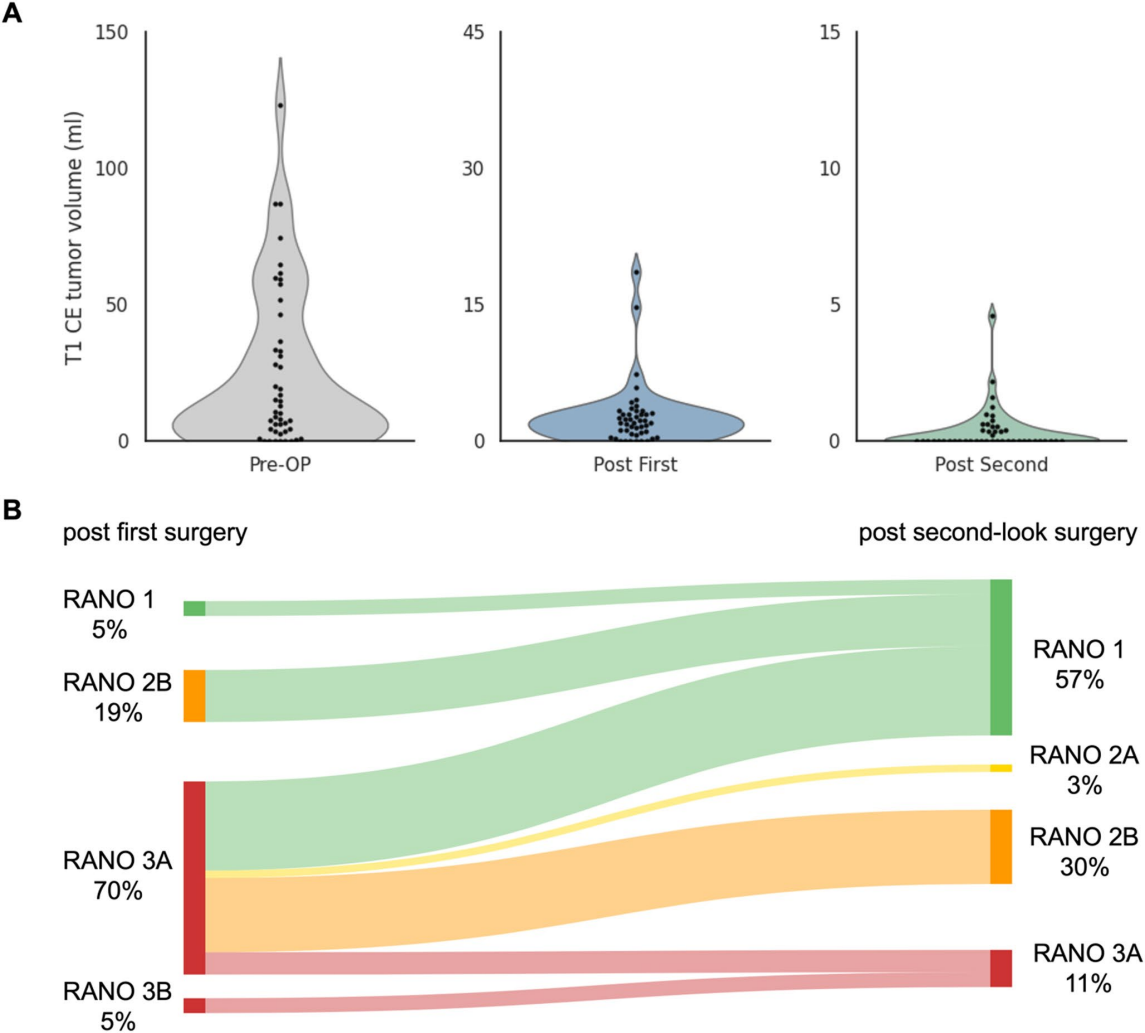
Tumour volumes and change in RANO class from first to second-look surgery in GBM patients. **A**: Violin plots display the distribution of T1-CE tumour volumes (ml) pre-operatively, after the first surgery and after second-look surgery. The black dots represent single patients, **B**: Sankey diagram mapping the proportion of patients in the respective RANO resect class after first surgery and the class after second-look surgery. Ribbon width is proportional to the number of patients; colours correspond to the destination class

In this cohort, the mean residual CE or nCE tumour was 2.67 cm^3^ (± 0.51) or 1.97 cm^3^ (± 0.47) after the first surgery and 0.367 cm^3^ (± 0.119) or 0.55 cm^3^ (± 0.19) after second-look surgery (Fig. 2A). Resection results of patients diagnosed with GBM (*n* = 37) after the first surgery corresponded to RANO resect class 1 in 5.4% (*n* = 2), 2B in 18.92% (*n* = 7), 3A in 70.27% (*n* = 26) and 3B in 5.4% (*n* = 2) (Fig. 2B). Resection results changed from 80.43% submaximal resection after the first surgery to 86.96% supramaximal and maximal resection after the second surgery. Second surgery significantly reduced residual tumour volume of both T1-CE (Wilcoxon signed-rank test, Z = − 5.44, *p* < .001) and T2-nCE (Z = − 5.37, *p* < .001) tumour. After the second surgery, resection results in the GBM group corresponded to RANO resect class 1 in 56.76% (*n* = 21), 2A in 2.7% (*n* = 1), 2B in 29.73% (*n* = 11), 3A in 10.81% (*n* = 4). Importantly, the proportion of RANO class 1 resections in the GBM subgroup increased from 5.4% after the first surgery to 56.8% following second-look surgery.

All IDH-mutant tumours in this cohort (*n* = 9) showed residual tumour volume > 1 cm^3^ (T2-nCE) after the first surgery. After the second surgery, complete resection was achieved in five of nine IDH-mutant tumours. Residual tumour volume > 1 cm^3^ was revealed in three patients and < 1 cm^3^ in one patient.

GBM patients in whom second-look surgery failed to achieve supramaximal or maximal resection (residual T1-CE > 1 cm³, RANO class 3, *n* = 4) already exhibited significantly larger residual CE and nCE tumour volumes after the first procedure (T1-CE, *p* = .045; T2-nCE, *p* = .031). To explore potential risk factors for failing to achieve complete CE-resection after second-look surgery, we performed a subgroup analysis comparing patients with no residual T1-CE tumour (RANO class 1–2 A, *n* = 22) to those with any remaining contrast-enhancing tumour (RANO class 2B or 3, *n* = 15). Patients with residual T1-CE tumour after second-look surgery had significantly larger postoperative T1-CE volumes following the first procedure (*p* = .023). Preoperative tumour size and functional characteristics including eloquent location, motor or speech involvement, and hemispheric distribution, did not differ significantly between groups (all *p* > .1).

### Functional and neurological outcome

**Fig. 3:**
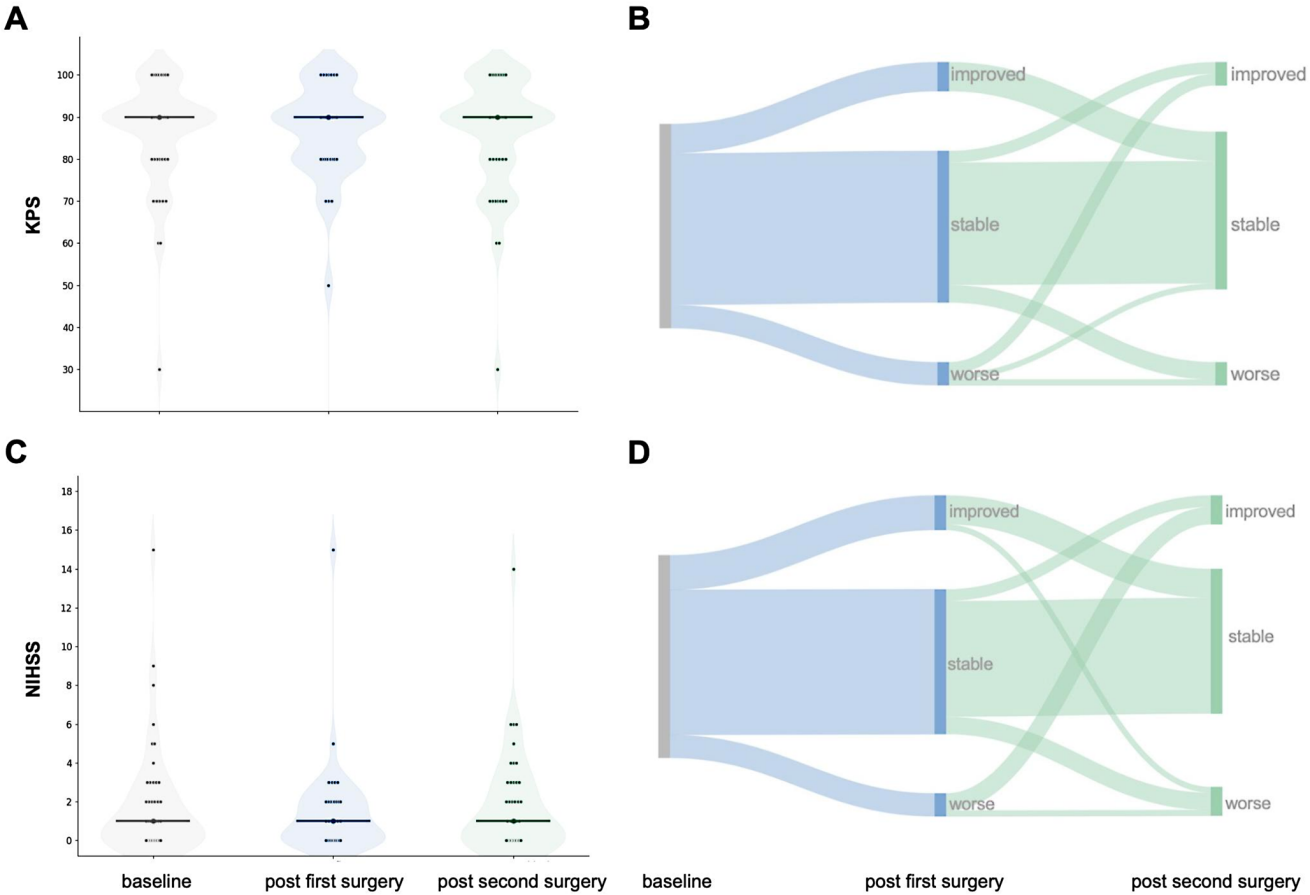
Functional and neurological performance over the course of surgeries. **A**: Violin plots depict the distribution of KPS at three time points: pre-operative baseline, post-first surgery, and post-second-look surgery. Individual patient values are overlaid; central markers indicate summary tendency (median and spread), **B**: corresponding Sankey diagram showing within-patient transitions in relative KPS (improved, stable, worse) from baseline to post-first surgery to post-second-look surgery. Ribbon width is proportional to the number of patients following each trajectory. **C**: Violin plots for NIHSS at the same time points, with individual observations overlaid. **D**: Corresponding Sankey diagram for relative NIHSS changes across the same time points

Functional and neurological outcome analyses include the entire cohort (*n* = 46), comprising both GBM and IDH-mutant tumours. The median postoperative KPS was 90% (range 50–100) and the median NIHSS was 1 (range 0–15) after the first surgery. Median Charlson comorbidity index before first surgery was 3 (range 2–6). Postoperative new neurological deficits occurred in 10.86% (*n* = 5) of patients with mainly speech and motor deficits or general clinical deterioration after the first surgery and in 6.52% (*n* = 3) after the second surgery. Yet, there was no significant change in functional and neurological status between postoperative assessments after the first and second surgery (KPS: Z = − 0.59, *p* = .557; NIHSS: Z = – 0.57, *p* = .569). Peri- and postoperative complications of the second surgery occurred in five (10.87%) cases, requiring surgical intervention under general anaesthesia (Clavien-Dindo grade IIIb) in four cases (8.7%) with two wound infections, one CSF leakage, and one postoperative bleeding. 30-day mortality was 0%. Figure 3 shows perioperative functional (KPS) and neurological (NIHSS) status as well as trajectories from baseline to first and second-look surgery.

### Survival analysis

**Fig. 4:**
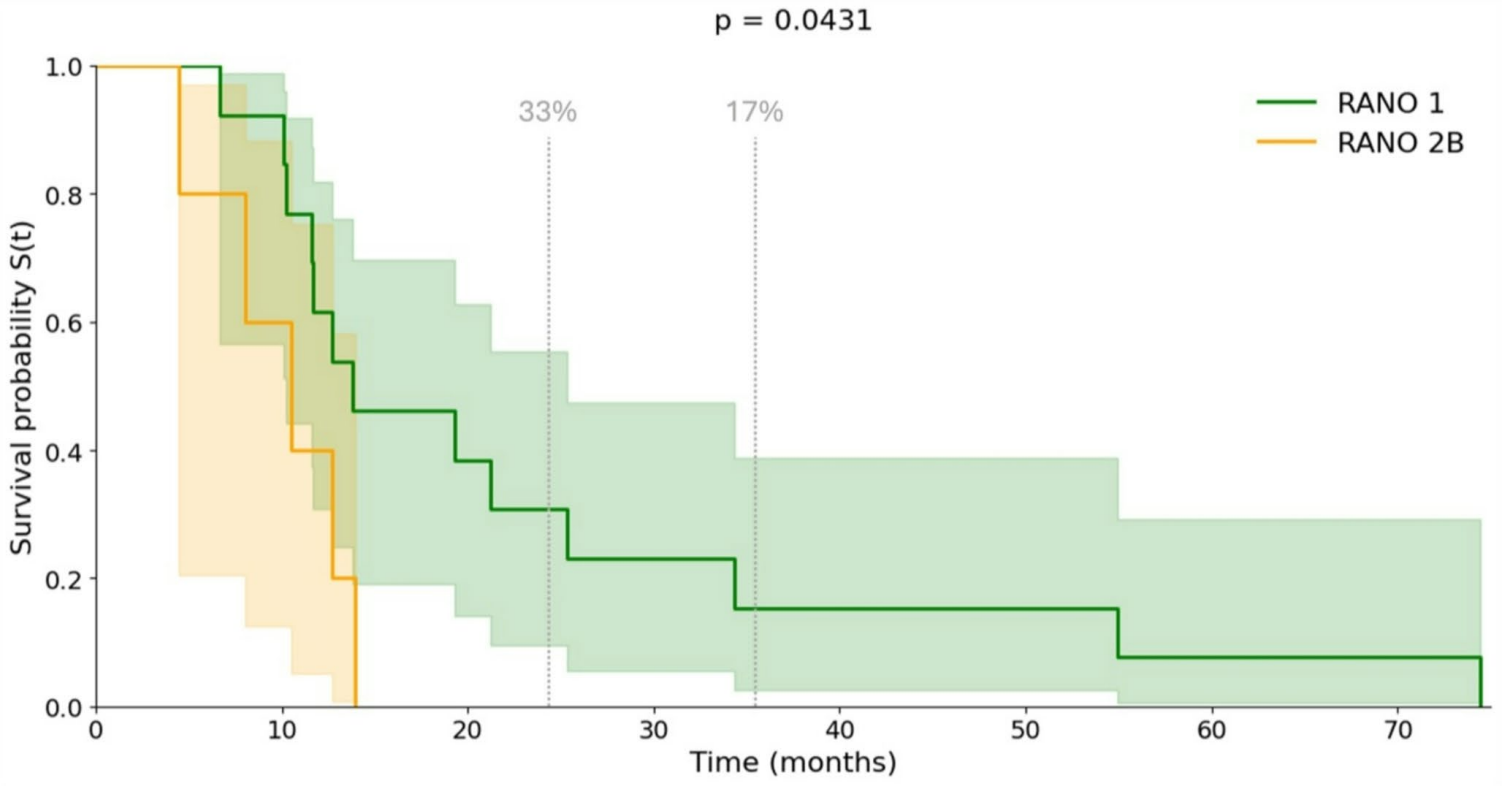
Kaplan–Meier analysis of overall survival according to RANO class after second-look surgery. Kaplan–Meier survival curves illustrate the overall survival of newly diagnosed GBM patients stratified by RANO resect class (*n* = 28). Patients without residual CE tumour (RANO class 1, green curve) demonstrated a significantly longer mOS compared to those with ≤ 1 cm^3^ residual tumour (RANO 2B, orange curve) (13.78 months (95% CI: 12.32–36.2) versus 8.03 months (95% CI: 1.98–15.92); log-rank test, *p* = .0431). The x-axis represents survival time in months, and the y-axis depicts the estimated survival probability S(t). The coloured bands surrounding both curves denote the 95% CI. The grey dashed lines indicate the percentage survival of patients in RANO class 1 after 2 and 3 years (33% and 17%, respectively)

In the subgroup of patients with newly diagnosed GBM (*n* = 28), median PFS was 9.3 months (95% CI: 7.76–10.82), with estimated 2-year and 3-year PFS rates of 14.3% and 9.52%, respectively. Median OS was 11.6 months (95% CI: 7.11–16.15), with 2-year and 3-year OS rates of 27.78% and 16.67%. According to RANO resect class after second-look surgery, patients in RANO class 1 reached a median PFS of 9.33 months (95% CI: 6.73–11.96) compared to 6.1 months (95% CI: 0.40–11.77) in RANO class 2B. The difference was not statistically significant (log-rank *p* = .329). Two- and three-year PFS rates were 16.67% and 8.34% for RANO class 1, while no patients in RANO class 2B group remained progression-free at 2 or 3 years. For OS, RANO class 1 patients showed a significantly longer median OS of 13.78 months (95% CI: 12.32–36.2) versus 8.03 months (95% CI: 1.98–15.92) in RANO class 2B (log-rank *p* = .043). The 2- and 3-year OS rates were 33.34% and 16.67% in RANO class 1, while no patients in RANO class 2B group survived beyond 2 years (Fig. 4).

A comprehensive overview of the study cohorts’ results is presented in Table 1.

**Table 1:** Summary of demographic, clinical, imaging, surgical, and outcome characteristics of the cohort (*n* = 46). Demographics include age, sex distribution, and the proportion undergoing second-look surgery after glioma surgery at first diagnosis versus at recurrence, as well as the interval between procedures. Tumour-related variables cover intraoperative assessment of completeness of resection at first and second surgery; anatomic localisation (hemispheric and lobar distribution; bilateral, multifocal, and deep-structure involvement); CNS WHO grade and molecular markers (e.g., IDH, 1p/19q, MGMT, where available). Quantitative tumour burden is reported as MRI-derived volumes (cm^3^; mean ± SEM) on pre- and postoperative scans (contrast-enhanced T1-weighted and T2/FLAIR), including residual tumour volumes for IDH-mutant gliomas. Surgical strategies are detailed for both operations (awake vs. asleep procedures, intraoperative neuromonitoring, and use of fluorescence guidance and IONM). Clinical status includes Charlson comorbidity index (median, range); KPS and NIHSS scores pre- and postoperatively for each surgery; pre-existing neurological deficits by domain and new postoperative deficits; and perioperative complications itemized and graded according to the Clavien-Dindo classification. Outcomes are presented as PFS and OS with medians and 95% confidence intervals, along with 2- and 3-year survival rates. PFS and OS are further stratified by RANO resection classes after second-look surgery. Unless otherwise specified, data are shown as means ± SD or SEM, or medians with ranges; percentages are calculated from available denominators

Patients (*n*)				46	
Demographics					
Age (years, mean, SD)				58.96 ± 13.07	
F: M ratio				1:1.09	
Second-look surgery (%)					
After initial glioma surgery				82.6	
After recurrence				17.4	
Mean time between surgeries (days, SD)				10.43 ± 6.38	
Intraoperative inspection shows complete tumour removal (%)					
First surgery				100	
Second-look surgery				87.5	
Localisation (n)					
Left hemisphere				19	
Right hemisphere Bilateral Frontal involvement Parietal involvement Temporal involvement Occipital involvement Periventricular Involvement of thalamus, insula, or basal ganglia Multifocal				25 2 23 7 14 12 0 3 6	
Tumour classification (n)					
CNS WHO grade 2				2	
IDH-mutant IDH-mutant + 1p/19q-codeleted CNS WHO grade 3 IDH-mutant IDH-mutant + 1p/19q-codeleted CNS WHO grade 4 IDH-mutant MGMT-methylated				1 1 7 5 2 37 0 17	
Adjuvant therapy for glioblastoma after first diagnosis (*n* = 28)				28	
TMZ only TMZ + Rx (*Stupp*) TMZ + Rx+CCNU (*Herrlinger*) Interval first surgery to start radio-chemotherapy (d, median, range)				1 25 2 37.5 (31.3–43.3)	
Clinical scores first surgery					
Charlson comorbidity index (median, range)				3 (2–6)	
Deficit pre-op (n)					
Motor Sensory Neglect Speech Vision/cranial nerves deficit Vigilance Behavioural changes Headache Seizure Deterioration of general condition Other				11 2 3 9 12 7 10 8 7 3 0	
Tumour volumes first surgery (cm^3^, mean, SEM)		Tumour volumes second surgery (cm^3^, mean, SEM)			
Pre-op T1-CE Pre-op T2-nCE Post-op T1-CE Post-op T2-nCE	25.06 ± 4.32 6.78 ± 1.19 2.67 ± 0.51 1.97 ± 0.47	Post-op T1-CE Post-op T2-nCE			0.37 ± 0.12 0.55 ± 0.19
RANO resect class (glioblastomas, n)	37	RANO resect class (glioblastomas, n)			37
1 2A 2B 3A 3B 4	2 0 7 26 2 0	1 2A 2B 3A 3B 4			21 1 11 4 0 0
Residual tumour volumes (IDH-mutant tumours, n) 0 cm^3^ < 1 cm^3^ > 1 cm^3^	9 0 0 9	Residual tumour volumes (IDH-mutant tumours, n) 0 cm^3^ < 1 cm^3^ > 1 cm^3^			9 5 1 3
Surgical strategy first surgery (n)		Surgical strategy second surgery (n)			
Awake	14	Awake			4
Asleep Intraoperative neuromonitoring ISIS Xpert C2 Xplore Fluorescence-guided surgery	30 43 20 23 40	Asleep Intraoperative neuromonitoring ISIS Xpert C2 Xplore Fluorescence-guided surgery			40 42 10 32 38
Clinical scores post first surgery		Clinical scores post second surgery			
New deficit post-op Motor Sensory Neglect Speech Vision/cranial nerves Vigilance reduced Behavioural changes Headache Seizure Deterioration of general condition Other	5 3 1 0 3 2 1 0 0 0 1 0	New deficits post-op (n) Motor Sensory Neglect Speech Vision/cranial nerves Vigilance reduced Behavioural changes Headache Seizure Deterioration of general condition Other			3 1 0 0 0 1 1 0 0 0 2 0
KPS pre first op (median, range) KPS post first op (median, range) NIHSS pre first op (median, range) NIHSS post first op (median, range)	90 (30–100) 90 (50–100) 1 (0–15) 1 (0–15)	KPS pre second op (median, range) KPS post second op (median, range) NIHSS pre second op (median, range) NIHSS post second op (median, range)			90 (60–100) 90 (30–100) 2 (0–5) 1 (0–14)
Complications of second surgery (n)			4		
Intra-op seizure			0		
Post-op seizure PAE intra-op PAE post-op Cardiac arrest intra-op Cardiac-arrest post-op Bleeding/infarction Hydrocephalus CSF leak Site infection/delayed wound healing			0 0 0 0 0 1 0 1 2		
Clavien-Dindo-Classification			5		
I			2		
II III IV V			0 3 0 0		
Survival in GBM after first diagnosis (n) PFS (months, median, 95% CI) 2-year PFS (%) 3-year PFS (%) OS (months, median, 95% CI) 2-year OS (%) 3-year OS (%)			28 9.33 (7.78–10.84) 14.3 9.52 11.67 (7.12–16.19) 27.78 16.67		
PFS according to RANO resect class post second-look (months, median, 95% CI)					
1			9.33 (6.73–11.96*)*		
2A 2B 3A			*n.a.* 6.1 (0.4-11.77) *n.a.*		
RANO 1					
2-year PFS (%) 3-year PFS (%)			16.67 8.34		
RANO 2B					
2-year PFS (%) 3-year PFS (%)			0 0		
OS according to RANO resect class post second-look (months, median, 95% CI)					
1			13.78 (12.32–36.2)		
2A 2B 3A			*n.a.* 8.03 (1.98–15.92) *n.a.*		
RANO 1					
2-year OS (%) 3-year OS (%)			33.34 16.67		
RANO 2B					
2-year OS (%) 3-year OS (%)			0 0		

Abbreviations: CE, contrast-enhancing; CI, confidence interval; CNS WHO, Central Nervous System World Health Organization classification; FLAIR, fluid-attenuated inversion recovery; IDH, isocitrate dehydrogenase; KPS, Karnofsky Performance Status; MGMT, O6-methylguanine-DNA methyltransferase; MRI, magnetic resonance imaging; NIHSS, National Institutes of Health Stroke Scale; OS, overall survival; PFS, progression-free survival; RANO, Response Assessment in Neuro-Oncology; SD, standard deviation; SEM, standard error of the mean

## Discussion

In this retrospective single-centre study, we analysed patients diagnosed with diffuse gliomas who underwent unplanned early second-look surgery due to residual tumour volume after assumed complete resection. The principal findings are that second-look operations (i) significantly reduced both CE and nCE residual volumes, shifting most patients into more favourable RANO resect classes, (ii) resection results were achieved without relevant neurological or functional deterioration, and (iii) in GBM, achieving supramaximal resection after the second surgery translated into a measurable OS advantage versus near total resection. Notably, long-term survivors were observed only among patients who reached RANO class 1 after the second surgery, underlining the prognostic importance of complete resection in this setting.

The discrepancy between intraoperative impression of complete resection and postoperative MRI findings is a frequent dilemma. Although, ioMRI allows real-time assessment of residual tumour, Roder et al. demonstrated that its routine use does not increase the proportion of true complete resections on early postoperative MRI after intraoperative imaging nor the rate of complete CE-resections when compared with 5-ALA fluorescence-guided surgery [14]. A national audit in the United Kingdom demonstrated that complete resection was assumed in more than 80% of cases, but confirmed on early MRI in less than half, while early reoperation was rarely pursued, mostly due to perceived lack of clinical benefit [25]. Data from our study group that assessed mapping and monitoring techniques in patients with supratentorial malignant lesions, also demonstrated that in 14% [15], respectively 16% [16] of the procedures, an unexpected residual tumour volume was seen in the postoperative MRI.

Our results extend prior, smaller series on early reoperation. Schucht et al. demonstrated the feasibility of early 5-ALA-guided redo surgery in nine patients, achieving complete resection without new neurological deficits [26]. Troya-Castilla et al. reported 11 patients undergoing early reoperation with complete removal archived in 58.62% and improved PFS and OS compared with a matched non-reoperated cohort without additional functional compromise [27]. Adding to these reports, our series includes 46 patients reoperated for residual tumour, underscoring that this strategy is implementable at scale in routine care.

Complete removal of CE tumour is a well-established prognostic factor in GBM [1], and increasing evidence supports the additional relevance of nCE resection [3, 4]. In our cohort, an intended complete resection was achieved in 86.96% of patients after the second surgery. RANO resection classification provided meaningful prognostic stratification, with class 1 resections associated with longer median OS compared to class 2B (13.78 months versus 8.03 months, *p* = .043). Survival beyond 2- and 3-years was observed exclusively in class 1, reinforcing the oncological relevance of complete CE tumour resection even when achieved through re-intervention.

Residual resection status after second-look surgery was primarily determined by the magnitude of postoperative CE and nCE residual volumes after the first surgery. In contrast, preoperative tumour volume, eloquent localisation, neurological status, and perioperative complications did not significantly predict resection class. A subset of 10.8% of GBM patients remained in RANO class 3 A, and three of nine patients with IDH-mutant tumours had residual volumes > 1 cm³ after re-intervention. These cases warrant consideration, yet also intraoperative impression during the second surgery suggested complete resection in only 87.5% using 5-ALA fluorescence-guided surgery and ioUS. Residual volumes were small, and all patients remained clinically stable without new deficits, suggesting patient-specific functional rather than technical and imaging-related limitations.

We also focused on clinical deterioration after the second surgery in our analysis, since new postoperative neurological deficits can offset much of the survival gain achieved with complete resection [28, 29]. In our cohort, functional deterioration occurred in five patients. Only one developed a new neurological deficit, while others experienced worsening of pre-existing symptoms, suggesting that deterioration often reflects worsening of pre-existing rather than new deficits. Importantly, all five showed improved resection results, illustrating the need to balance oncological gain against individual function.

Furthermore, second-look surgery did not critically delay initiation of adjuvant therapy. The median interval between first surgery and start of radio-chemotherapy was 37.5 days, well below thresholds associated with inferior outcomes [30]. This finding supports integration of early re-resection into multimodal treatment pathways when residual disease is identified.

Despite accumulating evidence, early second-look surgery remains underutilised. A global survey of neurosurgeons reported second-look procedures in < 1% of GBM cases, with decisions strongly influenced by tumour location and molecular features [31]. Together with national audit data [25], this underscores the gap between surgical practice and emerging evidence. Our results provide real-world data supporting early second-look surgery as a feasible and safe option to optimise the extent of resection when residual tumour is detected on early postoperative imaging.

## Limitations

This study has several limitations. First, its retrospective single-centre design introduces inherent selection bias, as patients undergoing early second-look surgery likely represent a favourable and selected subgroup. Second, the overall number of patients undergoing reoperation was limited (*n* = 46), with a predominance of glioblastoma cases (*n* = 37), restricting generalisability to other diffuse glioma entities. Third, assessment of residual tumour on early postoperative MRI may be influenced by reactive enhancement or postoperative changes. Fourth, heterogeneity in adjuvant treatment across the study period and the small number of IDH-mutant tumours limited molecular subgroup analyses. Finally, the absence of a matched non-reoperation control group precludes definitive conclusions regarding causal survival benefit.

## Conclusion

In conclusion, unplanned second-look surgery due to residual tumour volume as revealed in postoperative MRI in patients with diagnoses of diffuse glioma is feasible, safe, and improves resection result without delaying adjuvant therapy. Our data highlight that long-term survival in GBM patients was achieved exclusively in those who reached supramaximal resection after the second surgery, underscoring that maximising the extent of resection remains a priority. Immediate second-look surgery should be considered a viable strategy to achieve this goal, and prospective multicentre studies are needed to define selection criteria, integrate advanced intraoperative imaging, monitoring, as well as molecular diagnostics, and clarify survival effects within molecularly defined subgroups.

## Data Availability

The data supporting the findings of this study are not publicly available. The ethical approval granted by the local ethics committee does not cover data sharing or transfer to third parties. Therefore, the data cannot be made available to protect patient confidentiality and to comply with ethical and legal requirements.
